# Percutaneous, transpapillary, and transmural drainage in acute cholecystitis: a comparative analysis of techniques, stent selection, and clinical

**DOI:** 10.1097/MS9.0000000000003527

**Published:** 2025-07-30

**Authors:** Zim Warda Hasan, Tusneem Shehzadi, Anmol Mohan, Asad Ur Rehman, Fnu Muskan, Mariam Zia, Priyanka Mohan Lal, Hasibullah Aminpoor, Hasiba Karimi, Vikash Kumar, Usha Tejwaney, Sarwan Kumar

**Affiliations:** a Clinical and Translational Science, School of Medicine, West Virginia University, Morgantown, WV, USA; b Department of Medicine, University College of medicine and dentistry, Karachi, Pakistan; c Carle Foundation Hospital, Urbana, IL, USA; d Independent Medical College Faisalabad, Faisalabad, Punjab, Pakistan; e Jinnah Sindh Medical University, Karachi, Pakistan; f Akhtar Saeed Medical and Dental College, Lahore, Pakistan; g Ziauddin University, Karachi, Pakistan; h Kabul University of Medical Sciences, Kabul, Afghanistan; i Bezmialem Vakif University, Faculty of Medicine, Istanbul, Turkey; j The Brooklyn Hospital Center, USA; k Valley Health System, New Jersey, USA; l Wayne State University, USA

**Keywords:** acute cholecystitis, clinical outcomes, endoscopic gallbladder drainage, gallbladder drainage

## Abstract

Acute cholecystitis is a common inflammatory condition of the gallbladder, primarily caused by gallstone obstruction, leading to increased intraluminal pressure, ischemia, and infection. While laparoscopic cholecystectomy remains the standard treatment, gallbladder drainage techniques have emerged as effective alternatives for high-risk surgical patients. This review explores the indications, techniques, and comparative outcomes of various gallbladder drainage methods, including percutaneous transhepatic gallbladder drainage (PTGBD), endoscopic transpapillary gallbladder drainage (ETGBD), and endoscopic ultrasound-guided gallbladder drainage (EUS-GBD). Endoscopic ultrasound-guided transmural gallbladder drainage, particularly with lumen-apposing metal stents (LAMS), has shown high clinical success rates with fewer complications compared to traditional methods. Advances in stent technology and procedural techniques continue to improve patient outcomes. However, further research is needed to establish long-term efficacy and determine optimal patient selection criteria for different drainage methods. The review highlights the evolving landscape of gallbladder drainage and the need for standardized guidelines to optimize treatment strategies for high-risk patients.

**HIGHLIGHTS**

- This study is one of the few to directly compare percutaneous, transpapillary, and transmural drainage techniques for acute cholecystitis, providing a clear overview of their clinical outcomes, technical success, and complication profiles.
- It highlights the critical role of stent selection—specifically the superiority of LAMS in EUS-guided drainage—in influencing success rates and minimizing reinterventions.
- The findings offer practical guidance for selecting the most appropriate drainage technique based on patient risk and institutional expertise, aiding in the personalized management of acute cholecystitis.

## Introduction

Acute cholecystitis is primarily an inflammatory condition of the gallbladder, often caused by gallstone obstruction, leading to increased intraluminal pressure, ischemia, and infection^[1]^. The pathophysiology involves disturbances in gallbladder mucosal functions, with prostaglandins playing a significant role^[2]^. Clinical manifestations include right upper quadrant pain, fever, and nausea, with diagnosis typically confirmed via ultrasonography^[3]^. Risk factors for gallstone formation include obesity, age, and dietary habits^[3]^. Acute cholecystitis can also arise from non-gallstone-related causes, such as ischemia and infections^[4]^. While the mortality rate is generally low, it can be higher in elderly patients or those with acalculous cholecystitis^[4]^. Early intervention, particularly laparoscopic cholecystectomy, is crucial to prevent complications^[1]^.

Gallbladder drainage is an alternative to cholecystectomy for patients unfit for surgery due to high surgical risk^[5]^. Three main techniques are available: endoscopic transpapillary gallbladder drainage (ETGBD), endoscopic ultrasound-guided gallbladder drainage (EUS-GBD), and percutaneous gallbladder drainage (PCGBD)^[5]^. Endoscopic ultrasound-guided transmural gallbladder drainage has shown promising results, with higher clinical success rates compared to ETGBD and PCGBD^[5]^. Studies have demonstrated that EUS-GBD is as effective as PCGBD but with fewer adverse events and unplanned admissions^[6]^. Endoscopic ultrasound-guided transmural gallbladder drainage can be performed as a primary intervention for acute cholecystitis or to internalize drainage in patients with indwelling percutaneous cholecystostomy catheters^[7]^. The use of lumen-apposing stents in EUS-GBD has shown potential as a definitive treatment modality^[8]^. However, the optimal device and technique for EUS-GBD are yet to be determined^[7]^.

Endoscopic gallbladder drainage techniques have emerged as effective alternatives to percutaneous cholecystostomy for managing acute cholecystitis in high-risk surgical patients ^[9,10]^. Two main approaches are ETGBD and EUS-GBD^[11]^. Endoscopic ultrasound-guided transmural gallbladder drainage with lumen-apposing metal stents (LAMS) has expanded treatment options, enabling cholecystoscopy-guided interventions and serving as a rescue drainage method in malignant biliary obstruction^[9]^. Studies suggest that endoscopic techniques may be associated with lower adverse event rates and improved quality of life compared to percutaneous drainage^[11]^. However, the choice of drainage method depends on patient characteristics and technique availability^[10]^. While early studies showed promising results for endoscopic approaches^[12]^, further research is needed to establish their long-term efficacy and safety^[11]^.

## Indications and patient selection for gallbladder drainage

Gallbladder drainage is an alternative treatment for acute cholecystitis in high-risk surgical patients^[10]^. Various techniques exist, including percutaneous transhepatic gallbladder drainage (PTGBD), endoscopic approaches, and EUS-GBD^[13]^. While PTGBD is the most common method, endoscopic techniques are gaining popularity^[14]^. Compared to PTGBD, endoscopic gallbladder drainage (EGBD) has shown similar technical and clinical success rates, with fewer adverse events, shorter hospital stays, and reduced need for repeat interventions^[14]^. Endoscopic ultrasound-guided transmural gallbladder drainage, in particular, has demonstrated potential as a definitive treatment for surgically unfit patients, offering similar effectiveness to PTGBD with lower complication rates^[8]^. These drainage techniques can serve as a bridge to cholecystectomy or as definitive treatment in non-surgical candidates^[13]^.

The Tokyo Guidelines for gallbladder drainage in acute cholecystitis have evolved significantly, particularly with the 2018 updates. Percutaneous transhepatic gallbladder drainage is recommended as the first alternative to surgery for high-risk patients, especially those with moderate (grade II) or severe (grade III) cholecystitis^[15–17]^. For grade II patients, PTGBD should be considered if conservative treatment fails, while grade III patients require intensive care alongside PTGBD^[17]^. Additionally, endoscopic techniques such as transpapillary drainage and ultrasound-guided drainage are viable options in high-volume centers with skilled endoscopists^[16]^. The guidelines emphasize the need for further randomized trials to validate these drainage methods and their clinical effectiveness^[17]^. Overall, the updated guidelines provide a comprehensive framework for managing gallbladder drainage in acute cholecystitis^[18]^.

Gallbladder drainage is an alternative treatment for acute cholecystitis in high-risk surgical patients^[10]^. Percutaneous transhepatic gallbladder drainage is the most common method, but alternatives include percutaneous aspiration, endoscopic naso-gallbladder drainage, and gallbladder stenting^[13]^. Endoscopic ultrasound-guided gallbladder drainage is a newer technique requiring further evaluation^[10,13]^. Endoscopic approaches are particularly beneficial for patients with ascites, coagulopathy, or anatomically challenging cases^[19]^. The Tokyo Guidelines 2018 provide severity-based treatment strategies, recommending early laparoscopic cholecystectomy for mild cases and considering patient factors like the Charlson Comorbidity Index and ASA physical status^[19]^. Endoscopic gallbladder drainage offers internal drainage without percutaneous catheters, making it appealing for non-surgical candidates with symptomatic gallbladder disease^[20]^.

Key indications, techniques, and clinical factors for selecting appropriate candidates for gallbladder drainage are summarized in Table 1.

**Table 1:** Patient Selection

Aspect	Details	Reference
Indications for gallbladder drainage	Alternative treatment for acute cholecystitis in high-risk surgical patients	^[10]^
Techniques	PTGBD, endoscopic approaches (EGBD, EUS-GBD), percutaneous aspiration, endoscopic naso-gallbladder drainage, gallbladder stenting	^[13]^
Percutaneous transhepatic gallbladder drainage (PTGBD)	Most common method, recommended first for high-risk patients, especially in Grade II and III cholecystitis	^[15–17]^
Endoscopic gallbladder drainage (EGBD)	Similar success rates as PTGBD, but with fewer adverse events, shorter hospital stays, and reduced need for repeat interventions	^[14]^
Endoscopic ultrasound-guided gallbladder drainage (EUS-GBD)	Promising alternative with lower complication rates, suitable as definitive treatment for surgically unfit patients	^[8]^
Tokyo guidelines 2018	Recommend PTGBD for Grade II if conservative treatment fails, mandatory for Grade III with intensive care. Endoscopic techniques preferred in high-volume centers.	^[15–17]^
Patient selection factors	Consideration of Charlson Comorbidity Index, ASA physical status, and presence of ascites, coagulopathy, or anatomical challenges	^[19]^
Endoscopic drainage benefits	Provides internal drainage without percutaneous catheters, suitable for non-surgical candidates with symptomatic gallbladder disease	^[20]^
Need for further research	Randomized trials required to validate clinical effectiveness of newer drainage techniques	^[17]^

## Percutaneous Transhepatic Gallbladder Drainage (PTGBD)

Percutaneous transhepatic gallbladder drainage is an effective treatment for acute cholecystitis in high-risk patients, with technical success rates of 78–96%^[21,22]^. It serves as an alternative to surgery or percutaneous transhepatic biliary drainage, offering comparable outcomes for obstructive jaundice^[22]^. However, PTGBD has limitations, including catheter-related complications (0–25%) such as dislodgement, leakage, and blockage^[23]^. To address these issues, conversion to endoscopic ultrasound-guided gallbladder drainage (EUS-GBD) has been explored, showing high technical (92%) and clinical (90%) success rates^[24]^. While PTGBD allows for subsequent laparoscopic cholecystectomy, conversion rates to open surgery can be high (50%)^[21]^. PTGBD remains a safe and effective option for managing acute cholecystitis in high-risk patients, with potential for conversion to EUS-GBD in select cases.

## Endoscopic Transpapillary Drainage (ERCP-Guided Drainage)

Endoscopic ultrasound-guided biliary drainage (EUS-BD) and endoscopic retrograde cholangiopancreatography biliary drainage (ERCP-BD) are both effective techniques for managing malignant biliary obstruction, with comparable technical and clinical success rates^[25,26]^. Endoscopic ultrasound-guided biliary drainage may be associated with lower reintervention rates compared to ERCP-BD^[25]^. For acute cholecystitis in high-risk surgical patients, endoscopic gallbladder drainage techniques, including EUS-guided and transpapillary approaches, show promising outcomes compared to percutaneous cholecystostomy^[11]^. However, EUS-BD may carry a higher risk of cholangitis and stent migration compared to ERCP^[27]^. The choice between these techniques should consider patient-specific factors and requires a multidisciplinary approach^[11]^. While both methods demonstrate similar efficacy and safety profiles, further high-quality randomized trials are needed to establish definitive recommendations for their use as first-line treatments^[25,26]^.

## Endoscopic Ultrasound-Guided Transmural Gallbladder Drainage (EUS-GBD)

Endoscopic ultrasound-guided transmural gallbladder drainage is emerging as a viable alternative for patients unsuitable for surgery or percutaneous drainage. Studies have shown high technical and clinical success rates, ranging from 93% to 98.4%^[28–30]^. Long-term outcomes are promising, with excellent stent patency rates and low recurrence of acute cholecystitis^[28,30]^. Complications are relatively rare but can include pneumoperitoneum, bile peritonitis, and stent migration^[31]^. Endoscopic ultrasound-guided gallbladder drainage offers advantages such as internal drainage and the ability to perform therapeutic maneuvers via cholecystoscopy^[31]^. Various stent types have been used, including self-expandable metallic stents and LAMS, with good results^[28,29]^. Endoscopic ultrasound-guided gallbladder drainage appears to be a safe and effective option for gallbladder decompression in high-risk patients, potentially serving as a definitive treatment in some cases.

Table 2 compares the main drainage techniques (PTGBD, ERCP-guided transpapillary drainage, and EUS-GBD), highlighting their success rates, limitations, and typical hospital stays.

**Table 2:** Comparison of Gallbladder Drainage Techniques

Technique	Indications and Success Rates	Advantages	Limitations	Mean Length of Stay	Reintervention Rate	References
Percutaneous Transhepatic Gallbladder Drainage (PTGBD)	Effective for acute cholecystitis in high-risk patients; success rate: 78–96%	Alternative to surgery, allows subsequent laparoscopic cholecystectomy	Catheter-related complications (0–25%), high conversion rate to open surgery (50%)	7–10 days	20–30%	^[21–23]^
Endoscopic transpapillary drainage (ERCP-guided drainage)	Used for malignant biliary obstruction; comparable success rates with EUS-BD	Lower reintervention rates than percutaneous methods	Higher risk of cholangitis and stent migration compared to ERCP	6–9 days	10–15%	^[25–27]^
Endoscopic ultrasound-guided transmural gallbladder drainage (EUS-GBD)	High technical (93–98.4%) and clinical success rates	Internal drainage, long-term stent patency, allows therapeutic interventions	Rare complications: pneumoperitoneum, bile peritonitis, stent migration	5–7 days	5–10%	^[28–31]^

## Stent selection in EUS-guided transmural drainage

Endoscopic ultrasound-guided transmural gallbladder drainage is an effective technique for managing gallbladder issues in non-surgical candidates. Lumen-apposing metal stents have shown high technical (93%) and clinical success rates (100%) in EUS-GBD, with minimal adverse events^[29]^. A novel twisted fully covered self-expanding metal stent (SEMS) with spiral coiled ends has demonstrated feasibility in animal studies, offering potential advantages over existing stent types^[32]^. For pancreatic fluid collections, EUS-guided drainage is highly effective, with success rates ranging from 88.2% to 100% depending on the type of collection. Single-stent drainage appears to be as effective as multiple-stent drainage, with no significant difference in clinical success rates (93.9% vs 97.4%) or secondary infection rates (18.4% vs 5.3%)^[33]^. These findings suggest that stent selection and number may not significantly impact treatment outcomes in EUS-guided drainage procedures. Studies also suggest that both LAMS and novel SEMS designs can be effective for EUS-guided transmural drainage procedures, with the choice of stent depending on specific clinical scenarios.

## Technical and procedural considerations

Endoscopic ultrasound-guided gallbladder drainage has emerged as a safe and effective alternative for managing acute cholecystitis in high-risk patients^[28,34]^. The procedure typically involves transduodenal or transgastric access using LAMS or traditional biliary stents^[29,35]^. Technical success rates are high, ranging from 93% to 98.4%^[28,29]^. Clinical success is also favorable, with long-term stent patency rates of 86% at 3 years^[28]^. However, complications such as stent migration, occlusion, and bile leakage can occur^[28,35]^. Proper patient selection, expert endosonographers, and availability of surgical and radiological backup are crucial for optimal outcomes^[34]^. As the technique evolves, improvements in EUS accessories and dedicated stents are expected to enhance safety and expand indications for EUS-GBD^[34]^.

## Comparative analysis of drainage techniques

Gallbladder drainage techniques are essential for managing acute cholecystitis and biliary obstruction, particularly in high-risk patients who are not candidates for surgery. The primary techniques include PTGBD, endoscopic transpapillary drainage (ERCP-guided drainage), and EUS-GBD. Percutaneous transhepatic gallbladder drainage is highly effective, with success rates of 78–96%, and serves as an alternative to surgery, though it has catheter-related complications (0–25%) and high conversion rates to open surgery (50%). Endoscopic retrograde cholangiopancreatography biliary drainage-guided drainage is commonly used for malignant biliary obstruction, offering lower reintervention rates compared to percutaneous methods, but it is associated with a higher risk of cholangitis and stent migration. Endoscopic ultrasound-guided transmural gallbladder drainage is an emerging alternative with high technical (93–98.4%) and clinical success rates, providing internal drainage with long-term stent patency and the ability to perform therapeutic interventions such as cholecystoscopy, though rare complications like pneumoperitoneum, bile peritonitis, and stent migration can occur. Stent selection plays a crucial role in EUS-GBD, with LAMS and SEMSs demonstrating high efficacy, while novel SEMS designs are showing promise in experimental studies. Future advancements in stent technology and procedural techniques will continue to refine these approaches, improving outcomes and broadening indications for minimally invasive gallbladder drainage.

Table 3 provides a detailed side-by-side comparison of PTGBD, ERCP-guided (transpapillary) drainage, and EUS-GBD, including each method’s success rates and common complications.

**Table 3:** Comparison of Gallbladder Drainage Techniques

Technique	Indications and Success Rates	Advantages	Limitations
PTGBD	Acute cholecystitis in high-risk patients; Success: 78–96%	Alternative to surgery; allows laparoscopic cholecystectomy	Catheter-related complications (0–25%), high conversion to open surgery (50%)
ERCP-Guided Drainage	Malignant biliary obstruction; Success comparable to EUS-GBD	Lower reintervention rates	Higher risk of cholangitis, stent migration
EUS-GBD	High success rates (93–98.4%)	Internal drainage, long-term stent patency, allows therapeutic interventions	Rare complications: pneumoperitoneum, bile peritonitis, stent migration

## AI-driven advancements in gallbladder drainage and treatment precision

Recent advancements in artificial intelligence (AI) have opened new possibilities in medical procedures, including gallbladder drainage. Artificial intelligence has shown promise in improving the precision of drainage technique selection by integrating multimodal data such as imaging, laboratory indexes, and patient history. For example, AI models have been used to predict complications and suggest optimal treatment plans tailored to individual risk profiles, potentially reducing the reliance on operator experience and subjective judgment. Techniques like real-time ultrasound image analysis and puncture pathway planning are being developed to assist in complex procedures like EUS-GBD, potentially shortening the learning curve and making these procedures more accessible in primary care settings. Furthermore, AI-assisted real-time monitoring during the procedure can help prevent complications such as stent displacement or perforation by providing early warnings, while postoperative AI models can predict risks based on patient characteristics. This approach could not only standardize the selection of techniques but also reduce complications and improve overall patient outcomes.

The potential of AI in acute cholecystitis treatment was highlighted in studies such as the use of the AlphaFold model for molecular biology and drug discovery^[36]^, and AI models using clinical images for genomic prediction in cancer treatment^[37]^. These studies demonstrate the power of AI to transform patient care by providing individualized, data-driven recommendations. By integrating these AI capabilities into gallbladder drainage, we may be able to redefine the treatment paradigm for acute cholecystitis, promoting a more precise, effective, and equitable healthcare approach.

## Special considerations in drainage techniques

Gallbladder drainage techniques have evolved as alternatives to cholecystectomy for high-risk surgical patients with acute cholecystitis. Percutaneous transhepatic gallbladder drainage (PTGBD) remains the most common method, with high technical success rates^[5,13]^. Endoscopic approaches, including ETGBD and EUS-GBD, have emerged as viable alternatives in skilled hands^[15]^. Endoscopic ultrasound-guided gallbladder drainage has shown higher clinical success rates compared to PTGBD and ETGBD, while PTGBD maintains the highest technical success rate^[5]^. Endoscopic methods may offer lower adverse event rates and improved quality of life compared to percutaneous approaches^[11]^. However, the choice of drainage technique should consider patient characteristics, anatomical factors, and institutional expertise. Further, research is needed to establish the optimal approach for different patient populations^[11,13]^.

## Future directions and emerging technologies in

Recent advancements in gallbladder drainage (GBD) techniques for acute cholecystitis offer alternatives to early cholecystectomy, especially for high-risk patients. Percutaneous transhepatic gallbladder drainage remains the most common method, with high technical and clinical success rates^[17]^. Endoscopic approaches, including transpapillary and transmural techniques, have shown promise. Endoscopic nasogallbladder drainage and gallbladder stenting via transpapillary approach demonstrate good success rates but face technical challenges^[12]^. EUS-guided transmural GBD has emerged as a viable option, offering internalized drainage^[38]^. A network meta-analysis comparing percutaneous, endoscopic transpapillary, and EUS-guided GBD found that EUS-guided and percutaneous methods had the highest likelihood of technical and clinical success. EUS-guided GBD showed the lowest risk of recurrent cholecystitis, while percutaneous GBD had the highest risk of reintervention and unplanned readmissions^[39]^.

## Conclusion

Gallbladder drainage techniques have revolutionized the management of acute cholecystitis in high-risk surgical patients, offering viable alternatives to early cholecystectomy. Percutaneous transhepatic gallbladder drainage remains a widely used approach due to its high technical success rates, but endoscopic techniques, particularly EUS-GBD, have demonstrated superior clinical outcomes with fewer adverse events. The integration of advanced stents and novel procedural approaches continues to enhance the effectiveness and safety of these interventions. Despite promising advancements, challenges remain in optimizing patient selection, standardizing procedural techniques, and establishing long-term outcomes. Future research should focus on large-scale randomized trials to compare different drainage methods and refine clinical guidelines. By leveraging technological advancements and multidisciplinary expertise, the field of gallbladder drainage will continue to evolve, ultimately improving patient care and treatment success rates.

## Data Availability

Not applicable for this review.
